# Making US public health a good idea again

**DOI:** 10.1016/j.lana.2026.101423

**Published:** 2026-02-26

**Authors:** Toomas Timpka, Elin A. Gursky, James M. Nyce

**Affiliations:** a Department of Health, Medicine, and Caring Sciences, Linköping University, Linköping, Sweden; b Regional Executive Office, Region Östergötland, Linköping, Sweden; c College of Osteopathic Medicine, William Carey University, Hattiesburg, MS, USA; d Department of Anthropology, Ball State University, Muncie, USA

**Keywords:** Public health, United States, Sweden, COVID-19, Pandemic planning, Social contract, Vaccination, Systems, Illness, Innovation

## Abstract

The stress test the COVID-19 pandemic imposed on the US public health system illuminated predictable yet surprisingly unplanned for fault lines. A perceived lack of choice associated with nonpharmaceutical and pharmaceutical interventions led many Americans to question both measures and processes for mitigating disease consequences, such as masking and mass vaccination. A cultural-historical examination shows that a central impediment for US efforts to control the pandemic was the limited sense of common good. Many factors and beliefs, including also that the scientific-biotechnological innovation system did not serve the interests of all people equally, and the public health community's equating disease with how people perceived illness, weakened vaccination acceptance and disease control efforts. We conclude that US public health must renegotiate the social contract with the American people to recover a shared understanding of its relevance and to effectively respond to future health challenges and pandemics.

## Introduction

In 2020, the world was crippled by a seemingly once-in-a-century pandemic, contributing to some 7–20 million deaths. This pandemic became a stress test of America's public health systems. Americans in large part vilified the pandemic virus and welcomed a rapidly developed and distributed vaccine. However, six months after its introduction, the enemy had become the vaccine, not the virus, taking down with it trust in America's public health institutions. This shift from appreciation to resistance of pharmaceutical and nonpharmaceutical disease mitigation efforts can be understood by looking through a historical and comparative lens at public health in America. Winslow described in 1920 “the public health campaign” as the “science and art of preventing disease, prolonging life, and promoting health through the organized efforts of society (p. 30)”.^1^ After-action reports of the pandemic response produced by federal, state, and local public health agencies, academic institutions, nonprofits and independent commissions point out organizational failures also noted from previous crises that had threatened the health and lives of Americans.^2^ However, these reports do not illuminate why so many Americans this time turned against the vaccine and public health disease mitigation efforts.

We offer in this article explanations why human agency, digital age epidemiology, and the governance structure need attention if public health is to become an enterprise trusted and embraced by Americans in the next quarter of the 21st century. Approaching the nation's semiquincentennial, we consider the development of US public health systems as a backdrop which informed the recent pandemic response. A comparative lens is used to identify current challenges required to (re)position US public health as a “good idea” once again.

## The public health system in America

Early immigrants from Europe and Africa came to North America having experienced diseases and plagues, like the Black Death. Often transmitted through human-driven vectors, outbreaks of smallpox, cholera, and typhoid fever followed with them. The outbreaks were met by the public health tools available at the time, quarantine and isolation, which were implemented by health committees also responsible for improving water supplies and sanitation. As a sign of things to come, the development of a smallpox vaccine in 1796 interrupted the annual toll of transmissible deaths.^3^

The Industrial Revolution of the 19th century brought many rural groups into the cities. Denser populations in housing and worksites exacerbated the transmission of communicable diseases, like tuberculosis. The main measures available to protect populations from disease at the time were the provision of clean water, improvements in waste disposal and sewer systems, and increased food safety. The use of quarantines to separate the sick from the well was the long-established, and often the only tool for local infectious disease control. As time went on, protection of the population's health grew more formalized through the introduction of local and county health departments. Efforts became increasingly strained by the end of the century by the growing, national interconnectedness of road and rail that allowed for more rapid and efficient spread of communicable diseases. Additionally, enlarging its scope from communicable diseases to issues of worker protection, child labor, and other societal health issues, public health actions seemed, to some, to threaten the country's economic and industrial growth.^4^ Still, by the end of the 19th century, public health writ large remained a good idea for most Americans.

In the 20th century legislation like the Social Security Act of 1935 funded hospitals, declared war on poverty, and incentivized health care to the underserved.^5^ Vaccines and antibiotics developed through synergies across the government, universities, and pharmacological companies reduced the incidence of diphtheria, mumps, measles, and polio and increased lifespan and quality of life. An increasingly robust federal health apparatus also promoted national efforts aimed at injury reduction through automobile and motorcycle laws and addressed preventable morbidity through activities like water fluoridation and better food labeling. School entrance requirements, and buy in from the country's pediatricians, led to childhood vaccination rates attaining around 80% in the US.^6^^,^^7^ Federal funding also helped finance well baby and mother clinics which, too, increased healthy birth outcomes. As a result of these investments, life expectancy increased from approximately 47 to 77 years of age between 1900 and 2000, and infant mortality dropped from around 100 deaths per live births to about 7 per 1000 by 2000.^8^

Employer-sponsored health insurance was introduced during the Great Depression to help attract and retain workers, covering about 9% of Americans in the 1940's and 70% by 1960. But despite the expansion of health services like Medicare and Medicaid (and later the Affordable Care Act), public health agencies (and others like Federally Qualified Health Centers), have continued to serve as “a provider of last resort”, offering medical “safety net” services to some 34% of Americans.^9^ By the end of the 20th century, public health still remained a good idea, particularly for the chronically medically underserved.

The mission to prepare the nation for novel health threats was added to the public health agenda after the Second World War. Spurred by Cold War threats of biological warfare as well as the potential for pandemics, emerging and re-emerging pathogens, and weather-related catastrophes, federal funding in the fourth quarter of the 20th century and into the 21st century improved the *nation's* detection and response efforts^10^, ^11^, ^12^, ^13^, ^14^ given the *country's* patchwork of state and local health departments characterized by variabilities in resources and capabilities.^15^ Unfortunately, the federal pivot towards biodefense and homeland security strained the focus and funding that otherwise might have been directed to basic public health services.^16^ Nonetheless, the infrastructure built for vaccinating the youngest populations from childhood diseases, and adults from tetanus, Hepatitis B, and pneumococcal pneumonia, could be leveraged for the mass scale administration of medical countermeasures to protect Americans from deliberately deployed biological pathogens.

## A systems stress test

Leading up to the COVID-19 pandemic in 2020, data suggest that about 40% of Americans had a high level of trust in the US public health system.^17^ With pandemic vaccine development a priority initiated from the highest office in the country^18^ the mRNA COVID-19 vaccines were welcomed, even competed for, when first released. But that trust was soon put to the test across all aspects of vaccine acceptance and administration. Although the COVID-19 vaccine provided high rates of protection and reduced the risk of hospitalization and death after even one vaccination, many Americans considered any incidence of COVID disease a failure of the vaccine.^19^

The COVID-19 vaccination context was a novel experience. The medical “office visit” patient—provider model that most Americans equated with health care could not support the mass administration of the COVID-19 vaccines that necessitated security, verification, and large-scale cold chain requirements.^20^ COVID-19 vaccination required specific administration sites (like sports centers), incentivized monetary or other strategies, drove long waiting lines, and welcomed the assistance and surge capacity of disciplined, uniformed resources like the national guard.^21^ Moreover, being vaccinated did not necessarily allow Americans to re-engage in their normal social and work routines. Masking, social distancing, designated food shopping hours, closures of schools and team sports, and other such measures seemed draconian and unfair. COVID-19 health and safety rules that included restrictions in mobility, interruptions to the flow of civil society, and other (perceived) threats to liberty, freedom of choice, and the right “to assemble” were perceived as encroachments on Americans’ civil rights and liberties.^22^ Confinements became seen as “lockdowns” rather than “cordon sanitaire”. Family members of those fatally ill saw themselves barred from hospital visitations and unable to grieve as families or to participate in traditional burial services. The resulting loss of autonomy and dignity experienced by many Americans seemed inversely proportionate to the threat they perceived. All this diminished public health credibility and its efforts to carry out COVID-19 disease mitigation strategies.

## Four challenges illuminated

The stress test the COVID-19 pandemic aroused magnified both the strengths and weaknesses of the public health system's ability to protect Americans. It is noteworthy that the main theme in the pandemic experience is not the achievement of rapidly producing efficient and safe vaccines. Instead, the dominant theme is frustration based on two mutually reinforcing rhetorics—one that questioned the legitimacy and authority of public health and the other that challenged both science and medicine. A corresponding reaction to COVID-19 public health efforts was absent in many other countries. Examining the stress test through a comparative cultural-historical lens, four challenges that American public health needs to address today can be seen. In the present article, Sweden is used for the comparison because in absence of constitutional foundations for strict government control during health emergencies, the Swedish population was asked to adapt their behavior voluntarily.^23^

### Challenge 1. The social contract

The tradeoff of individual autonomy versus community good in health matters has a long history in America. The controversy was resolved legally in 1905 when the U.S. Supreme Court ruled for the state in Jacobson vs Massachusetts. In that ruling, the police powers of the state to protect the public's health were preserved over the rights of the individual to refuse vaccination for smallpox.^24^ Still, in America, acting for the “common good” continues to be seen as a threat to individual autonomy and choice,^25^ while such reasoning makes little cultural sense in many other countries. In Sweden, the pandemic response was not laissez-faire, as it often was described in the US,^26^ but leveraged by an incremental shift in perspective on health that reflected Sweden's history and legislation. Swedish policymaking carries a tradition of attempting to find common ground (and a common good) across society.^27^ This separates Swedish and American communities in fundamental ways. A few decades into the 20th century, Swedish industry and trade union leaders, scientists, and elected officials initiated cooperative programs for urban planning, sanitation, affordable housing, and public transportation to serve the common good.^28^ Adapting a British model of social medicine, Swedes also argued that social factors can be disease determinants as important as biological factors^29^: “Disease processes can be initiated not only by adverse factors in the purely physical environment but also by adverse factors in the social environment. […] Moreover, many conditions, such as tuberculosis, venereal disease, and many mental ills illuminate how the individual has a responsibility back to the community (p. 127)”. From a social medicine perspective (which should not be confused with socialized healthcare), health is considered not only on the *individual* but also on a *community*, attending to factors ranging from the micro (biological) to the macro (the social). By the 21st century, a model for the provision and innovation of health services based on a social contract and the “common good” was well established in Sweden.^30^ A factor facilitating this was that discussions across the boundary between the state and civil society in Sweden did not have the same negative overtones as they had in the US because, over time, the two had become seen as inextricably bound to each other. In fact, current Swedish uses the same word “samhälle” for both society and state.^31^

This bind between civil society and state helps explain why Swedish communities could adopt population-based social distancing measures in the early phase of the pandemic. Although no formal “lockdown” was established, Swedes broadly accepted voluntary recommendations to maintain personal distance, work from home, and avoid public transportation and large gatherings.^32^ The fact that Sweden maintains universal healthcare, childcare, and retirement funding facilitated both staying home and working from home for employees.^33^ When the vaccination program in Sweden was initiated in late December 2020, the vaccines, offered free of charge, were mainly mRNA vaccines developed in the US through Operation Warp Speed. By October 2021, about 85% of the Swedish adult population had been vaccinated compared to 70% in the US.^34^^,^^35^ Overall, during the COVID-19 pandemic, the excess mortality in Sweden, as in the other Scandinavian countries, was notably lower than in the United States.^36^

### Challenge 2. Disease and illness

Why the mRNA vaccines and public health institutions came to be rejected by many Americans during the pandemic requires an understanding of the concepts of “disease” and “illness”.^37^ Disease refers to the biological processes (and the knowledge) involved in a specific pathology, i.e., the textbook understanding of pathology that holds no matter where it occurs. Illness, on the other hand, refers to how a particular society defines “disease” using a set of cultural, but not entirely biological, categories or understandings. Americans' understanding of COVID-19 was based on some biological understanding of what an infection is and how it is caused, although interpreted, understandably, through a particular cultural, historical, and social lens. In the US, there has also often been a moral dimension associated with who gets ill and from what illness.^38^ For example, the notion of “germ theory” was seen at work during the Cold War, but germs were associated with a particularly malignant, “immoral” ideology.^39^ More recently this equation of morality and disease has been found in lay reasoning about HIV.^40^ Also, during the COVID-19 pandemic, Americans conflated morality with disease and illness, especially in alternative explanations about where COVID-19 illness originated and how it was caused. The “refusal” of the scientific establishment to take such theories seriously (which made perfect sense to a large number of Americans) deepened the public's skepticism about orthodox science and public health,^41^ and facilitated the acceptance (and little criticism) of alternative treatment approaches like prescription of antiparasitic drugs normally used in veterinary medicine. In other words, that public health institutions did not recognize how disease was understood by many Americans opened the door for subsequent attacks on (and alternatives to) science, medicine and public health.^42^ Rather than dismiss this as due to some deficit in the American public (attributed to educational levels or ideology, etc.),^43^ it is important to acknowledge the COVID-19 quickly became its own media event drawing in spokespersons whose messages were able to exploit the personal experience and life histories of many Americans.

### Challenge 3. The innovation system

At the time of the pandemic, US public health and its collaboration across research, scientific, and policy domains had contributed to worldwide international respect for America's science and medical community. Through federal grants, state and local public health departments had, for decades, expanded efforts in areas such as health promotion, maternal and child health, medical services delivery, and disease investigation and outbreak control. However, the innovation of these services is less problem-based and incremental than in countries like Sweden.^30^^,^^44^ Rather, the American research and policy enterprise was grounded in a vision of capitalism and science as being beneficial and mutually symbiotic.^45^ At the time of the COVID-19 pandemic, the National Institutes of Health (NIH) was responsible for driving innovation in health services by financing research and academic medicine and supporting collaborations with the biotechnological industry. In effect, the NIH maintained a steady state “innovation ecosystem” for health services founded on the development of novel biotechnology.^46^ For example, the WHO estimates that global immunization efforts have saved at least 154 million lives over the past 50 years^47^; US’ robust biotechnology sector has been a key contributor to these efforts.^48^ However, not to be discounted is the profitable industrialization of such innovation.^49^

In May 2020, public-private partnerships of federal agencies, universities, and biopharmaceutical companies from this ecosystem were invited by the White House and challenged to develop COVID-19 vaccines through “Operation Warp Speed”.^50^ By December 2020, the FDA was able to issue Emergency Use Approval for mRNA vaccines produced by the program, which then led to policy and strategies to administer the vaccine at the state and local levels. Such development would have been more time consuming or even unlikely in Sweden where the market economy does not follow these same rules.

Still, a short time after their release, the mRNA vaccines had become an object of suspicion in America. Misinformation and conspiracy theories, e.g. ill-founded rumors about materials “hidden inside” the mRNA vaccines like tracking devices, made many Americans skeptical about both vaccines and the public health vaccine delivery system.^51^ This strategy was mediated by large groups unfamiliar with the components and processes involved in the innovation system for rapid infectious disease prevention.^52^ In a social landscape skewed toward e-commerce and the marketing of products, social media often showcased critics of vaccination guidelines who could offer their followers uninformed, but to many Americans reasonable, answers to complex public health problems. A virus of unknown origin and a novel vaccine developed in a partnership between the government, scientists, and the pharmacological industry created a perfect “alternate reality” in which biotechnological innovation was redefined as threats to ordinary people.^53^

### Challenge 4. The population model

How one intervenes in public health is influenced by the population model one uses. For a century, American innovation ecosystems and its public and private health institutions have assumed that applying biological disease models to human societies will accomplish what needs to be done.^54^ However, when there have been unanticipated consequences to the products developed and programs implemented, US health institutions have seldom reached down “far enough” to analyze how their model of Americans could influence the project's negative or positive outcomes.^55^ During the pandemic, public health's commitment to this biological disease model led to its failing to understand how and why the vaccine and the government-industry partnership became demonized.^56^ Indirectly, this reduced the possibility of achieving the desired outcomes of the vaccination program with regard to vaccination coverage. Pragmatic endpoints like these often turn out to be difficult to achieve when biological population models are used to represent human behavior in society.^57^ But why has this issue not received the attention it deserves? This is likely, as Schneider has observed, because much of what passes for models of society are derived from “common sense notions, the everyday premises of the culture in which … the scientist lives”^58^ In short, this conflation of population and society makes sense because it “derives directly and practically unaltered from [our own] ethnoepistemology” i.e., with how nature is believed to operate in the world.^59^

## Making public health a good idea again

The American pandemic experience shows that a society facing an emerging health threat is dependent both on its ability to organize response measures and on the extent to which foundational beliefs about how the world operates are shared. Although Americans seldom invoke anything like a social contract between the government and members of the public,^60^ we find historical evidence shows that public health initiatives has invoked cooperation, affiliation, and collaboration in America.

Recently, a National Academy of Medicine initiative presented a vision for modernizing the public health system.^61^ Their proposal focused on four organizational areas where improvements are needed: cross-sector collaboration, financing mechanisms with accountability for health outcomes, improving data systems, and building a ready workforce. Our examination advocates that beyond organizational improvements, fundamental issues that focus on the interaction with the American people needs to be prioritized if the public health system is to regain credibility and legitimacy.

### Human agency

Public health institutions must extend their representation of society to include social hierarchies and human agency so as to build models in which individuals and communities can recognize themselves and at least in part agree. The skepticism about science and technology that surfaced during the pandemic can be traced back to social movements and social trends that have questioned the role and legitimacy of elites in government, academia, and large corporations. These movements have rested on the assumptions that 1) there is an (inherent) conflict between “ordinary people” and a self-interested “immoral elite” and 2) this conflict is associated with a social order in which science and technology are the main drivers that can increase human welfare.^62^ The societal power of the movements, in America and elsewhere, is played out not only through formal institutional structures such as economic, legal, health, and educational systems, but also through informal institutions like family, casual social networks, and civic organizations. Studies of the pandemic response have disclosed that the lack of attention to these informal structures, on the one hand, and the association between conceptions of illness and moral values held by many Americans on the other, made the “elite” dominated public health innovation system an open target for populist attacks.^63^ The populist rhetoric extended moral sanctions not just to disease itself but to the government institutions and biotechnological companies that contributed to Covid-19 vaccine administration and, by extension, to orthodox science and public health.^64^ What happened during COVID-19 suggests that public health must acknowledge and represent more accurately all factual social hierarchies in the American society and the human agency associated with these. Were public health institutions to represent the social order more accurately in intervention planning, they would facilitate disease prevention and mitigation efforts more closely aligned with how Americans think about health and disease and incorporate the cultural and historical understandings that underlie how these operate. For example, Americans may believe in some biological theory but, at the same time, hold theories or “agendas” that are at odds with how science understands vaccines and disease. Based on their personal experiences, they may fear that vaccines contain substances the authorities use to, e.g. monitor or control them. When deciding “what to do next”, many Americans may therefore turn to alternative theories because these appear to confirm and better reflect their construct of how the world works against them.

### Digital age epidemiology

Private companies and political interest groups in the US have learned to observe individuals and communities “from within”, tracking their conceptions of illness and related beliefs, moral values, emotional states, and behaviors, and leveraging these for marketing and product penetration. American public health institutions need to learn from these models to develop information age methods that can be used to identify new determinants of health and support diversified interventions.

The authority of public health coalesced when public health in the 19th and early 20th century classified individuals into social hierarchies derived from the labor market to inform housing, sanitation, and labor reforms.^4^ As America moves further into the 21st century, understanding the potential for disease prevention and health promotion will again require public health to represent the social order. However, in the interconnected digital society, the behaviors of individuals and groups are acquired not only from material conditions and traditional social obligations, but also from less tangible manipulation and/or monetization of self through various information channels,^65^ where the digital environment provides unprecedented possibilities to convey and impose viewpoints on others with significant consequences. The epidemiological methods used in public health need to be updated to become more valid, intelligible, and congruent with this new social/media landscape. In the coming decades, public health should look for methods to represent individuals and communities in social hierarchies not only originating from the labor market, but also from the information society at large, accounting for its association with human agency.^66^

### Nationally unified diversification of governance

Optimally, what should drive American public health agendas is a mutual recognition that good health and absence of disease empowers quality of life, reduces public expenditures, improves opportunity, and catalyzes economic growth. The system of public health institutions and agencies that has evolved since America's founding have been built to anticipate and respond to the needs of a growing nation, its global leadership, and robust economy. Present trends in American society will limit the ability of federal agencies to guide and fund the practice of public health through a national lens. In the immediate future, 50 governors, experiencing reduced federal funding, will determine how their states craft public health policy, provide public health services, and protect constituents from disease and disaster. As potential strategies, governors may elect to enlarge (and redefine) their programs through mutual assistance agreements with neighboring states, develop bulk purchasing contracts for pharmaceutical products, and/or collaborate with private sector delivery services to advance their public health agendas.

The views on disease and illness held in respective states, and the associated beliefs about their causes and treatments, have the potential to become a dividing force in the American population and to create an uneven health landscape. Conversely, health can also be a great unifier as many health conditions, like infectious diseases, are not constrained by geopolitical borders. Additionally, the country will have to decide how to sustain a nationally unified health security platform in the face of external threats and the next pandemic within this state-driven diversification (“patchwork”). Further, the present lack of shared trust and views on disease and illness will require diversification (and rewriting) of the social contracts between public health institutions and local communities. Such contracts will need to counterbalance the trend of self-guided health decision-making with efforts to incorporate the benefits of a common social contract.^67^ These efforts will take time and, in the short term, will result in parallel versions of everyday public health routines in which science and biotechnology may play different roles across the nation.

## Conclusions

The second quarter of the 21st century offers a unique opportunity to reconstruct a public health system grounded in mutual respect, shared perspectives, and participatory growth. Seen through the lens of history and culture, the recent experiences tell us that “success” in public health has to include in its calculations perceived quality of life as well as the benefits accrued from biotechnological innovation. As much as addressing biomedical innovations, public health objectives need to respect diverse cultures and opinions, understand and collaborate with formal and informal groups and information channels, and support populism that does not divide but rather unites the nation's human agency in common projects. To achieve this, public health should strive to disseminate the benefits of a social contract in which health is an important goal for all Americans, their families, and their neighbors.

What we propose here is nothing revolutionary. Bringing the common good back to attention is only a call to return to what Winslow argued a century ago–a public health initiative that would best address America's health needs (Text Box 1). What is necessary, though, is to recognize that the current complexities in governance, unremitting instances of disease outbreaks and disasters, and variation in the public's acceptance of public health practices call for changes in public health's methodological toolbox. To write its next chapter, American public health will require rethinking its strategy to understand and collaborate with informal groups and novel information channels. If this is built into today's health care equation, US public health could well become (very) much more than just a good idea.

*Text Box 1* **Winslow's description in 1920 of the American public health physician's tasks.1**

“The physician in the public health field practises medicine but with a difference, in that the goal before his eyes is prevention as well as cure, and that he has always in view not merely the individual but the community as well. […] He must be much more than a physician in order to fulfil this task; for he must have a knowledge of bacteriology and sanitation, of health administration and statistics, above all of social relationships and social machinery which the curriculum of even the best medical schools can not attempt to supply (p. 30.)”

## Contributions

Conceptualisation TT, EAG, JMN; investigation TT, EAG, JMN; methodology TT, EAG, JMN; project administration TT, EAG, JMN; validation TT, EAG, JMN; writing—draft TT; writing—review & editing EAG, JMN. All authors had direct access and have verified the underlying data reported in the manuscript.

## Declaration of interests

TT is employed by Linköping University and Region Östergötland and holds research grants from the Swedish Research Council, Region Östergotland, and the Research Council of Southeast Sweden. He is also head of the medical commission at Swedish Athletics. JMN holds research grants from the Mississinewa Foundation.
